# Controlled direct synthesis of single- to multiple-layer MWW zeolite

**DOI:** 10.1093/nsr/nwaa236

**Published:** 2020-09-14

**Authors:** Jie-Qiong Chen, Yu-Zhao Li, Qing-Qing Hao, Huiyong Chen, Zhao-Tie Liu, Chengyi Dai, Jianbo Zhang, Xiaoxun Ma, Zhong-Wen Liu

**Affiliations:** School of Chemical Engineering, Northwest University, Chemical Engineering Research Center of the Ministry of Education for Advanced Use Technology of Shanbei Energy, International Science & Technology Cooperation Base of MOST for Clean Utilization of Hydrocarbon Resources, Xi’an 710069, China; School of Chemical Engineering, Northwest University, Chemical Engineering Research Center of the Ministry of Education for Advanced Use Technology of Shanbei Energy, International Science & Technology Cooperation Base of MOST for Clean Utilization of Hydrocarbon Resources, Xi’an 710069, China; School of Chemical Engineering, Northwest University, Chemical Engineering Research Center of the Ministry of Education for Advanced Use Technology of Shanbei Energy, International Science & Technology Cooperation Base of MOST for Clean Utilization of Hydrocarbon Resources, Xi’an 710069, China; School of Chemical Engineering, Northwest University, Chemical Engineering Research Center of the Ministry of Education for Advanced Use Technology of Shanbei Energy, International Science & Technology Cooperation Base of MOST for Clean Utilization of Hydrocarbon Resources, Xi’an 710069, China; Key Laboratory of Syngas Conversion of Shaanxi Province, School of Chemistry & Chemical Engineering, Shaanxi Normal University, Xi’an 710119, China; School of Chemical Engineering, Northwest University, Chemical Engineering Research Center of the Ministry of Education for Advanced Use Technology of Shanbei Energy, International Science & Technology Cooperation Base of MOST for Clean Utilization of Hydrocarbon Resources, Xi’an 710069, China; School of Chemical Engineering, Northwest University, Chemical Engineering Research Center of the Ministry of Education for Advanced Use Technology of Shanbei Energy, International Science & Technology Cooperation Base of MOST for Clean Utilization of Hydrocarbon Resources, Xi’an 710069, China; School of Chemical Engineering, Northwest University, Chemical Engineering Research Center of the Ministry of Education for Advanced Use Technology of Shanbei Energy, International Science & Technology Cooperation Base of MOST for Clean Utilization of Hydrocarbon Resources, Xi’an 710069, China; Key Laboratory of Syngas Conversion of Shaanxi Province, School of Chemistry & Chemical Engineering, Shaanxi Normal University, Xi’an 710119, China

**Keywords:** MWW, 2D zeolite, zeolite nanosheets, amphiphilic organosilane, alkylation

## Abstract

The minimized diffusion limitation and completely exposed strong acid sites of the ultrathin zeolites make it an industrially important catalyst especially for converting bulky molecules. However, the structure-controlled and large-scale synthesis of the material is still a challenge. In this work, the direct synthesis of the single-layer MWW zeolite was demonstrated by using hexamethyleneimine and amphiphilic organosilane as structure-directing agents. Characterization results confirmed the formation of the single-layer MWW zeolite with high crystallinity and excellent thermal/hydrothermal stability. The formation mechanism was rigorously revealed as the balanced rates between the nucleation/growth of the MWW nanocrystals and the incorporation of the organosilane into the MWW unit cell, which is further supported by the formation of MWW nanosheets with tunable thickness via simply changing synthesis conditions. The commercially available reagents, well-controlled structure and the high catalytic stability for the alkylation of benzene with 1-dodecene make it an industrially important catalyst.

## Teaser

The single- to fewer-layer ultrathin MWW zeolite with controlled structure, high crystallinity and excellent thermal/hydrothermal stability was facilely synthesized by using commercially available structure-directing reagents, which makes it an industrially important catalyst for converting bulky molecules.

## INTRODUCTION

Zeolites are microporous crystalline aluminosilicates with excellent thermal/hydrothermal stability, diverse pore topologies and tunable acidity in a wide range, which enable their extensive application in catalysis especially in petroleum refining [1–3]. However, zeolite catalysis is still challenged by the diffusion limitation and the thermal/hydrothermal stability of the materials [4–8]. To this end, a single-unit-cell nanosheet of zeolites is efficient to minimize the diffusion limitation and to expose more external acid sites [9–13]. Thus, the post-exfoliation of layered MWW zeolites [9,14–17] and the structure-directing agent (SDA) assisted direct synthesis [10,18–21] are commonly investigated to fabricate single-layer MWW (SL-MWW) zeolites. Among these routes, the direct synthesis is preferred because of its simple procedure, high solid yield, and more importantly the high structural integrity [22–27]. Although zeolites MCM-56 [17,28,29], ITQ-30 [30] and EMM-10 [31] with disordered MWW layers along the *c*-axis can be synthesized directly without the assisted SDA, the MWW layers are not fully delaminated to SL-MWW zeolites as revealed from the commonly lower external surface areas.

By using a rationally designed bifunctional SDA, Ryoo *et al*. pioneered the directly synthesizing single-unit-cell nanosheets of MFI zeolite [10]. With a similar strategy, the delaminated MWW zeolite nanosheets named MIT-1 are recently reported [18]. In addition, the delaminated MWW zeolite (DS-ITQ-2) containing a large proportion of MWW monolayers is directly synthesized by hexamethyleneimine (HMI)-assisted bifunctional SDAs [19]. Nevertheless, these specially designed SDAs requiring complicated synthetic procedure can only be used for a specific zeolite, which limits their potential industrial application [22–27,32]. Therefore, the readily available and affordable SDA is desirable for the controlled synthesis of ultrathin MWW nanosheets.

In essence, the common feature of previous works is that the functional groups of the bifunctional SDA molecules are embedded in the zeolite layers while the long alkyl chains prevent their stacking along the *c*-axis [22,23]. Indeed, the structure of the commercially available amphiphilic organosilane is very similar to that of specially designed SDA i.e. the methoxysilyl moiety can incorporate into MWW layers via covalent bonds with growing crystals while the bulky hydrophobic tail can restrict the ordered stacking of MWW layers along the *c*-axis. Although the strategy is seemingly simple, unfortunately only multilayered MWW nanosheets (15–60 nm) can be synthesized by using HMI and amphiphilic organosilane as dual-SDAs [33,34]. By using similar organosilane as co-SDA, comparable results are obtained in the cases for synthesizing Ti-MWW [35] and SAPO-34 zeolite nanosheets [36]. If these related reports are analyzed, one can find that changing the molar ratio of TPOAC/SiO_2_ and/or the chain length of the organosilane is commonly applied to tune the crystal size or the mesopore size of zeolite [6,33,36–38]. In contrast, the other key factor, i.e. the balance between the rates for the formation of the nanocrystals from the inorganic aluminosilicate precursor and the incorporation of the organosilane into the nanocrystals, is largely omitted. In fact, the rate for the crystallization of the inorganic aluminosilicate is generally higher than that of the organosilane in this dual-SDAs system. As a result, when inorganic aluminosilicates are used as silicon/aluminum sources, larger nanocrystals are expected to be rapidly formed at the initial stage of crystallization, which expels the organosilane from the aluminosilicate domain. Thus, the direct synthesis of the controlled and highly crystalized single-unit-cell nanosheets is still challenging, especially in cases using the amphiphilic organosilane as a co-SDA.

For the layered MWW zeolite, a recent non-local density functional theory (DFT) study reveals that the ordered staking of MWW layers of MCM-22 is mainly controlled by the inductive effects of HMI through forming strong hydrogen bonds with surface silanols [39]. Following this mechanism, the formation of multilayered zeolite structure in the dual-SDAs system is mainly determined by the competitive interaction between the HMI and naked MWW layers. Accordingly, to prevent the inductive effects of HMI, the co-SDA must be embedded in or grafted on every single MWW layer, each of which are formed at the initial stage of the synthesis. In contrast, the naked MWW layers will be assembled into multilayered structures providing the inductive effects of HMI. Thus, by simply balancing (i) the formation rate of the single-unit-cell nanosheet via the nucleation and growth of aluminosilicate with (ii) the speed for incorporating the organosilane into the single-unit-cell nanosheet, highly pure SL-MWW zeolites are reasonably expected. Almost simultaneously to our work, 2D MWW nanosheets with 1–2 unit cells are reported by just changing the amount of hexadecyltrimethylammonium bromide (CTAB) added into the synthesis gel of MCM-22 zeolite [40].

**Scheme 1.**
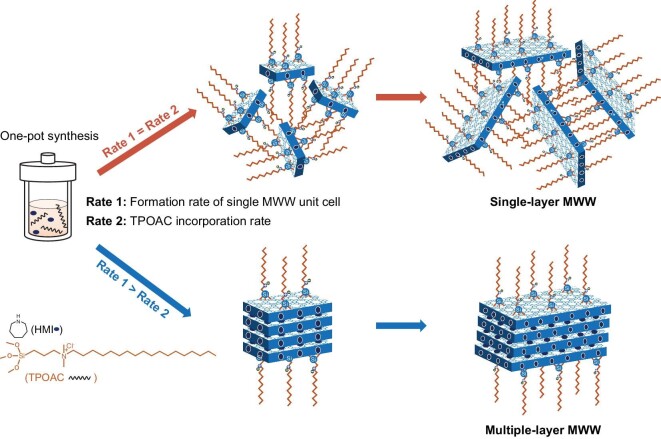
Illustrative scheme for the formation of single-layer and multiple-layer MWW zeolite.

Herein, using HMI and commercially available amphiphilic organosilane ([(CH_3_O)_3_SiC_3_H_6_N(CH_3_)_2_C_18_H_37_]Cl, TPOAC) as combinational SDAs, we demonstrate the controlled direct synthesis of the SL- to ML-MWW zeolite via regulating the rates for the formation of the single-unit-cell nanosheet and the incorporation of the organosilane. Following the proposed mechanism (Scheme 1), well-structured SL-MWW zeolite with excellent thermal/hydrothermal stability was obtained. Moreover, the thicknesses and arrangement of MWW layers could be well regulated by simply changing the concentration of alkalinity and HMI under a constant molar ratio of TPOAC/SiO_2_ in the synthesis gel (Scheme S1 in the online Supplementary Materials). Significantly, it is expected that this proposed mechanism could be used for realizing the direct synthesis of single-layer zeolites with different topologies.

## RESULTS AND DISCUSSION

### Synthesis of the SL-MWW zeolite

**Figure 1.**
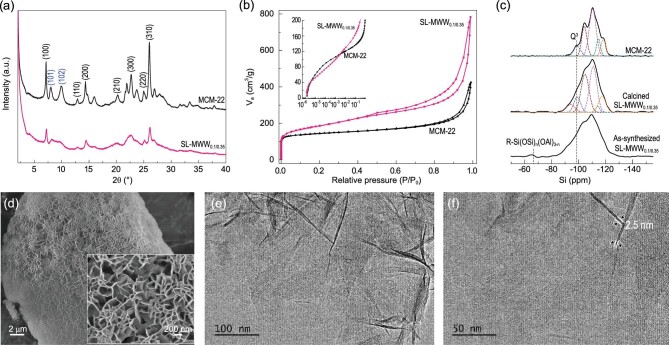
Structure of single-layer MWW zeolite. (a) XRD patterns and (b) N_2_ adsorption-desorption isotherms for SL-MWW_0.1/0.35_ and MCM-22; (c) ^29^Si MAS NMR spectra of as-synthesized SL-MWW_0.1/0.35_, calcined SL-MWW_0.1/0.35_ and MCM-22; (d) SEM and (e and f) TEM images of SL-MWW_0.1/0.35_.

The synthesis of a SL-MWW zeolite was demonstrated by using a synthesis gel of 0.1 Na_2_O/1 SiO_2_/0.033 Al_2_O_3_/0.35 HMI/0.04 TPOAC/45 H_2_O with 14 days crystallization (denoted as SL-MWW_0.1/0.35_, where 0.1 and 0.35 represent the molar ratios of Na_2_O to SiO_2_ and HMI to SiO_2_ in the synthesis gel, respectively). Powder X-ray diffraction (XRD) was used to identify the thickness and arrangement of MWW layers in the 2θ range of 6–10° [13,20]. As shown in Fig. 1(a), the MCM-22 presents two intense and discrete (101) and (102) reflections at 2θ of 8 and 10^o^, indicating the thick and ordered staking of MWW layers. For the zeolite MCM-56 (Fig. S1a), the (101) and (102) reflections are clearly visible although the peak valley is increased to a certain extent, which indicates the partial condensation of the adjacent MWW layers after calcination as explained in the references [29,39]. In the case of calcined SL-MWW_0.1/0.35_, the (101) and (102) reflections are transformed into a broad peak due to the disordered arrangement of single-unit-cell MWW nanosheets along the *c*-axis (Fig. 1(a)). Moreover, only the (*hk0*) reflections are sufficiently sharp for indexing for the diffraction pattern due to the smaller thickness along the *c*-axis, which is in good agreement with the previous simulated and experimental XRD patterns of MWW nanosheet with single-unit-cell thickness [18–21,41].

This can be further confirmed by the N_2_ adsorption-desorption results (Fig. 1(b)). Compared to the MCM-22, the adsorption isotherm of SL-MWW_0.1/0.35_ presents a sharp increase in uptake of nitrogen in the relative pressure ranges >0.9, indicating the presence of intercrystalline meso/macropores formed by the intergrown and ‘house of cards’ disposition of MWW layers. Moreover, the uptake of nitrogen for SL-MWW_0.1/0.35_ in the relative pressure ranges of 10^−6^ < P/P_0_ < 10^−3^ is lower than that for MCM-22 (the inset of Fig. 1(b)), indicating the absence of 12-MR supercages over the delaminated structure of SL-MWW_0.1/0.35_ [15,18,19]. The external surface area and pore volume of SL-MWW_0.1/0.35_ (446 m^2^/g and 1.21 cm^3^/g) are significantly increased compared to that of MCM-22 (Table 1). Figure 1(c) shows the ^29^Si magic-angle spinning nuclear magnetic resonance (MAS NMR) spectra of as-synthesized and calcined SL-MWW_0.1/0.35_. A clear signal at −65 ppm derived from R-Si(OSi)_n_(OAl)_3-n_ is detected for the as-synthesized sample, confirming that the organosiloxanes are connected to the framework or the surface of MWW layers [36,42]. After calcination, the resonance signal at −65 ppm is disappeared due to the removal of organic groups. Moreover, the Q^3^ (Si(OSi)_3_OH) resonances at about −100 ppm for calcined SL-MWW_0.1/0.35_ are significantly increased compared to that for MCM-22, which further confirms the single-layer structure with higher density of silanol due to the formation of 12-MR cups on the external surface.

**Table 1.** Textural properties of MCM-22, MCM-56 and ultrathin MWW zeolites.

	*S*_BET_	*S*_ext_ ^a^	*S*_ext_/*S*_BET_	*V*_total_	*V*_micro_ ^a^
Sample	(m^2^/g)	(m^2^/g)	(%)	(cm^3^/g)	(cm^3^/g)
					
MCM-22	550	119	22	0.66	0.17
MCM-56	448	183	41	0.75	0.13
ML-MWW_0.15/0.5_	532	248	47	0.75	0.14
ML-MWW_0.1/0.5_	595	417	70	1.03	0.09
SL-MWW_0.1/0.35_	640	446	70	1.21	0.09
ML-MWW_0.1/0.35/0.03_	465	164	57	0.83	0.10

^a^ *S*_ext_ (external surface area) and *V*_micro_ (microporevolume) calculated using *t*-plot method in the 3.5–5.0 Å thickness range.

The scanning electron microscope (SEM) image (Fig. 1(d)) shows that the SL-MWW_0.1/0.35_ prepared here presents an ultrathin flake-like morphology in a ‘house of cards’ disposition, which is very different to the MCM-22 and thick plate-like morphology of MCM-56 (Fig. S2). Transmission electron microscope (TEM) images (Fig. 1(e) and (f); Fig. S3) reveal that most of the crystals for the SL-MWW_0.1/0.35_ prepared here present a single MWW layer with single-unit-cell thickness (2.5 nm) along the (001) direction. However, MWW nanosheets with few bilayered structures (Fig. S3) in the SL-MWW_0.1/0.35_ are inevitable under the synthesis conditions used here. In addition, no amorphous form was detected in the SEM and TEM visualization.

### Mechanism of the ML- to SL-MWW zeolites

**Figure 2.**
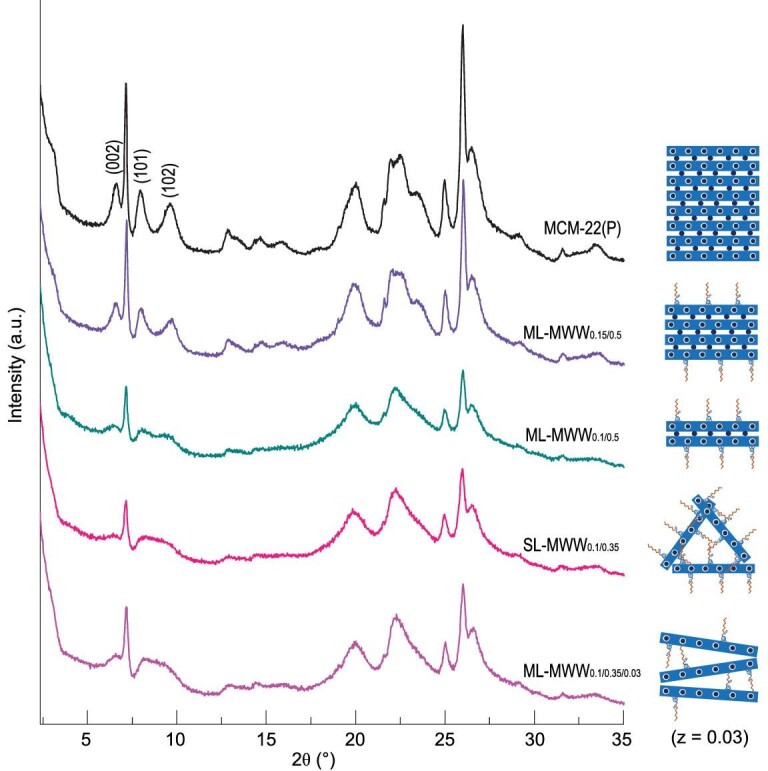
XRD patterns and proposed arrangement of MWW layers for the as-synthesized samples using a synthesis gel of *x* Na_2_O/1 SiO_2_/0.033 Al_2_O_3_/*y* HMI/*z* TPOAC/45 H_2_O.

The proposed formation mechanism of the MWW nanosheets in the dual-SDAs system can be verified by the fact that the thickness of MWW nanosheets could be tailored by regulating the ratio of either Na_2_O to SiO_2_ or HMI to SiO_2_ while keeping a constant concentration of TPOAC in the synthesis gel. As shown in Fig. 2, MCM-22(P), where *P* is for the MCM-22 precursor, presents a clear (002) diffraction peak and distinct (101) and (102) reflections at 2θ values of 8° and 10°, indicating a highly ordered staking of MWW layers. For the duel-SDAs system, using the same concentration of Na_2_O and HMI (*x* = 0.15 and *y* = 0.5) with that of MCM-22, only ML-MWW zeolite (denoted as ML-MWW_x/y_, where *x* and *y* represent the molar ratios of Na_2_O to SiO_2_ and HMI to SiO_2_ in the synthesis gel, respectively) can be obtained, which can be validated by the distinct (002) and discrete (101) and (102) reflections for the as-synthesized ML-MWW_0.15/0.5_ although the intensity is decreased (Fig. 2). It should be noted that the discrete (101) and (102) reflections emerge even after only six days crystallization (Fig. S4), indicating the formation of a multilayered structure at the early stage. However, an additional signal at −52 ppm derived from R-Si(OSi)_n_(OAl)_2-n_(OH) is present from the ^29^Si MAS NMR results (Fig. S5). This indicates that the condensation rate of the silanol in an organosilane molecule is lower than the formation rate of the single-unit-cell MWW nanosheet by inorganic silica and alumina source (Scheme S1b). As a result, the multi-layered MWW nanosheets are formed due to the inductive effects of HMI through forming strong hydrogen bonds with surface silanols, although the thickness of MWW nanosheets is decreased compared to the MCM-22(P). In contrast, if the ratio of Na_2_O to SiO_2_ is decreased (*x* = 0.10 and *y* = 0.50), the intensity of (002) peak obviously decreases and (101) and (102) peaks become weaker and broader for ML-MWW_0.1/0.5_ (Figs 2 and S6), suggesting that the thickness of MWW nanosheets is further decreased. This result can be reasonably explained by the decreased formation rate of the single-unit-cell MWW nanosheets, which is gradually matched with the incorporation rate of TPOAC (Scheme S1c). With an optimal concentration of Na_2_O and HMI (*x* = 0.10 and *y* = 0.35), the (002) diffraction peak of SL-MWW_0.1/0.35_ is very weak and is nearly invisible in the XRD patterns (Fig. 2). This clearly indicates that most of the crystals are single-layered structures although bi- or multi-layered structures cannot be ruled out. Moreover, the (101) and (102) reflections are transformed into a broad peak due to the disordered arrangement of the MWW layer grafted with organosilanes along the *c*-axis. These observations indicate that the rates between the nucleation/growth of the MWW nanocrystals and the incorporation of TPOAC into the single MWW unit cell was matched very well, the SL-MWW zeolite with ‘house of cards’ deposition was obtained (Scheme S1d). In addition, if the concentration of TPOAC in the synthesis gel is decreased (*z* = 0.03), the as-synthesized sample shows a clear (002) reflection and a broad peak in the 2θ range of 8–10° (Figs 2 and S7), which reflect the multilayered MWW nanosheets with vertically misaligned structure like UJM-1P [20]. This indicates that a minimum coverage of incorporated TPOAC is required for disoriented layers along the *c*-axis (Scheme S1e). This is further supported by the clearly lower S_ext_/S_BET_ of ML-MWW_0.1/0.35/0.03_ (57%) (Table 1) than that of SL-MWW_0.1/0.35_ (70%).

The evolution of MWW layer thickness when changing the composition of the synthesis gel was further proved by the N_2_ adsorption-desorption (Fig. S8 and Table 1), SEM and TEM (Fig. S9) results. As shown in Table 1, the external surface area of MCM-22 and MCM-56 is lower than that of ultrathin MWW zeolites prepared by using TPOAC as co-SDA. Significantly, the external surface area of ultrathin MWW zeolites is increased with decreasing the ratio of either Na_2_O to SiO_2_ or HMI to SiO_2_ in the synthesis gel (Table 1), which indicates that the thickness of MWW nanosheets is gradually decreased. Moreover, the almost identical S_ext_/S_BET_ for SL-MWW_0.1/0.35_ and ML-MWW_0.1/0.5_ indicates that few bilayer MWW nanosheets are coexisted with the prevailing single-layer MWW nanosheets over SL-MWW_0.1/0.35_, which is in good agreement with the XRD and TEM results. From SEM and TEM images of MCM-22 (Fig. S9a), the agglomerate morphology is observed with thick plates of MWW crystals. After the introduction of TPOAC to the synthesis gel of MCM-22, the ML-MWW_0.15/0.5_ exhibits plate-like morphology with more open disposition, but the mean thickness of the MWW nanosheets is about 10.0 nm (Fig. S9b). If the ratio of Na_2_O to SiO_2_ is further decreased (*x* = 0.1 and *y* = 0.5), the ML-MWW_0.1/0.5_ exhibits flake-like morphology with obvious intergrowth disposition of thinner MWW nanosheets (about 5.0 nm) (Fig. S9c). Indeed, 2D MWW nanosheets with 1–2 unit cells can be synthesized by just changing the concentration of CTAB in the dual-SDAs system [40]. If the synthesis process is analyzed, the work essentially supports our proposed mechanism, i.e. the decrease in formation rate of the MWW unit cell by increasing the concentration of CTAB results in CTAB being adsorbed on MWW nanosheets, which are formed at the initial stage of the synthesis. These results further confirm the proposed formation mechanism of the MWW nanosheets in the dual-SDAs system, i.e. the balanced rates between the nucleation/growth of the MWW nanocrystals and the incorporation of the organosilane into the MWW unit cell. Following this mechanism, the direct synthesis of MWW nanosheets with controlled thickness was achieved.

### Stability, acidity and catalytic performance of SL-MWW zeolite

**Figure 3.**
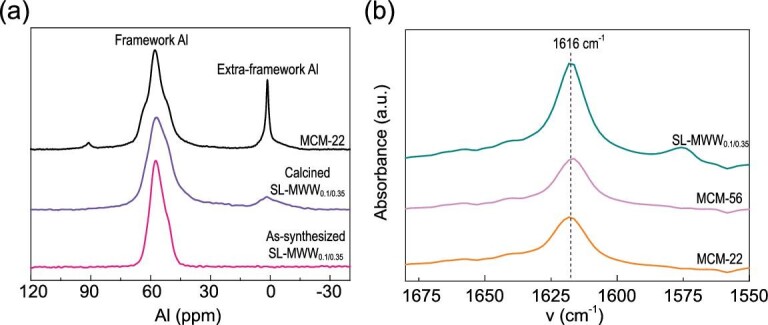
(a) ^27^Al MAS NMR spectra of Na-type as-synthesized and calcined SL-MWW_0.1/0.35_, and MCM-22; (b) FT-IR spectra of H-type MCM-22, MCM-56 and SL-MWW_0.1/0.35_ after adsorption DTBP.

The composition, stability and acidity of the SL-MWW_0.1/0.35_ synthesized here was further characterized. The SiO_2_/Al_2_O_3_ ratios of MCM-22 and SL-MWW_0.1/0.35_ is 23 and 29 (Table S1), respectively. Compared to MCM-22, the additional TPOAC was added into the synthesis gel for the preparation of SL-MWW_0.1/0.35_, resulting in a higher SiO_2_/Al_2_O_3_ ratio. Moreover, no amorphous phase was observable in the SEM and TEM images. Thus, the higher SiO_2_/Al_2_O_3_ ratio of SL-MWW_0.1/0.35_ can be reasonably attributed to the incorporation of TPOAC into the MWW layers. The thermal stability of the SL-MWW_0.1/0.35_ can be demonstrated by the ^27^Al MAS NMR spectra of as-synthesized and calcined sample (Fig. 3(a)). The proportion of aluminum species remaining in tetrahedral coordination for calcined SL-MWW_0.1/0.35_ is about 92%, which is significantly higher than that for MCM-22. This result indicates that the SL-MWW_0.1/0.35_ exhibited excellent thermal stability, which may be related to the lower crystallization rate of SL-MWW_0.1/0.35_. Moreover, the hydrothermal stability can be confirmed by the fact that approximately 90% of the initial tetrahedral Al is retained in the framework of Na-type SL-MWW_0.1/0.35_ even after being heated in 100% steam at 680°C (Fig. S10). The total and external acid site density was measured by NH_3_-TPD and FT-IR spectroscopy with di-*tert*-butyl-pyriding (DTBP) adsorption [43]. The NH_3_-TPD results (Fig. S11) show that the total acid site density over SL-MWW_0.1/0.35_ is lower than that over MCM-22 and MCM-56, which can be attributed to the higher SiO_2_/Al_2_O_3_ ratio of SL-MWW_0.1/0.35_ and the partly lost acidic site located in the 12MR supercage. But the external acid site density over SL-MWW_0.1/0.35_ is almost two times higher than that over MCM-22 and MCM-56 (Fig. 3(b) and Table S1). This result further confirms the disorder and open structure of SL-MWW_0.1/0.35_, and that a higher proportion of the acid sites is located on the external surface over SL-MWW_0.1/0.35_.

**Figure 4.**
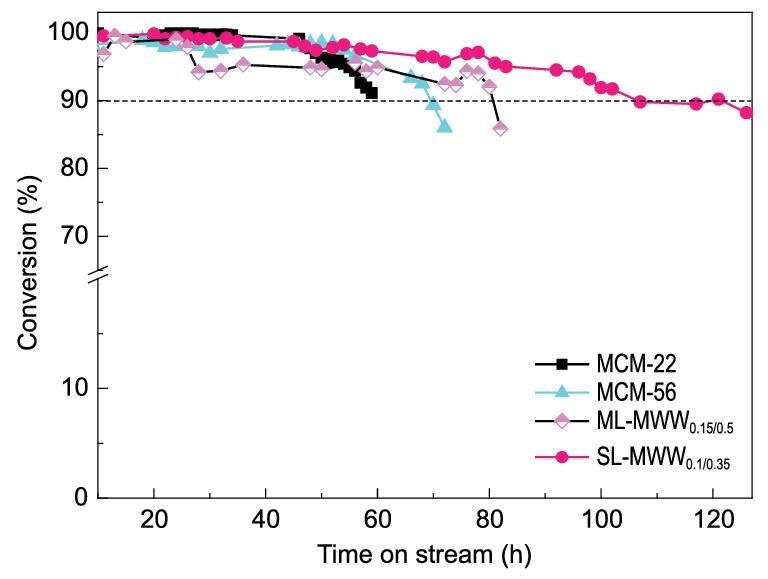
Time-on-stream conversion of 1-dodecene over the synthesized zeolites.

The Friedel-Crafts alkylation of benzene with long chain α-olefin is an industrial process for the preparation of linear alkylbenzene (LAB), which can be catalyzed by different zeolites with 12MR pores in the liquid phase [44]. However, the fast deactivation of the zeolite catalysts due to the diffusion limitation is currently a major hurdle for practical applications [45]. Thus, the alkylation of benzene with 1-dodecene was used as a model reaction to assess the catalytic activity and lifetime of SL-MWW_0.1/0.35_. The batch reaction results reveal that the conversion of 1-dodecene over SL-MWW_0.1/0.35_ (63%) and MCM-56 (65%) is almost similar, which is higher than that over MCM-22 (Table S2). It should be noted that the turnover number (TON) value (per acid site) over SL-MWW_0.1/0.35_ is almost twice that over MCM-22 and MCM-56 if the total acid sites are taken into account. This result is consistent with the fact that the number of external acid sites of SL-MWW_0.1/0.35_ is higher than that of MCM-22 and MCM-56. More importantly, as shown in Fig. 4, the SL-MWW_0.1/0.35_ is deactivated far more slowly than MCM-22, MCM-56 and ML-MWW_0.15/0. 5_ with the time on stream, which can be reasonably attributed to the decreased diffusion limitation over SL-MWW_0.1/0.35_ with single-layered structures [46].

## CONCLUSION

The concise SL-MWW zeolite with high thermal/hydrothermal stability was successfully synthesized by using the commercially available HMI and TPOAC as co-SDAs, and its high catalytic performance was demonstrated by the alkylation of benzene with 1-dodecene as a model reaction. Moreover, the mechanism for the formation of the SL- or ML-MWW zeolites, which depends on the rates for the formation of the single-unit-cell nanosheet directed by HMI and the incorporation of TPOAC, was rigorously revealed. Following the proposed mechanism, ultrathin MWW nanosheets with tunable thickness were obtained by simply changing the synthesis conditions. The commercially available reagents and easily controlled structure make the synthesis strategy very promising for a large-scale application. More importantly, the direct synthesis of single- to multiple-layer zeolites with varied topologies can be reasonably expected according to the proposed mechanism.

## METHODS

### Chemicals

Hexamethyleneimine (HMI, 99 wt%), colloidal silica (Ludox, AS-40, 40 wt% suspension in H_2_O) and dimethyloctadecyl[3-(trimethoxysilyl)propyl]ammonium chloride (TPOAC, 42 wt% in methanol) were purchased from Sigma-Aldrich. Sodium metaaluminate (NaAlO_2_, AR, 41 wt% Al_2_O_3_), sodium hydroxide (NaOH, AR), ammonium nitrate (NH_4_NO_3_, AR), benzene (AR) and 1-dodecene (AR) were purchased from Sinopharm Chemical Reagent Co. Ltd (China). All reagents were used as received without fruther purification.

### Synthesis of MWW zeolites

SL- to ML-MWW zeolites were prepared by using dual organic templates, specifically HMI as the SDA for directing the formation of MWW unit cells, and commercially available TPOAC as the assisted SDA for preventing their stacking along the *c*-axis. The TPOAC was mixed with colloidal silica, sodium aluminate, NaOH, HMI and distilled water, to give a gel molar composition of *x* Na_2_O/1 SiO_2_/0.033 Al_2_O_3_/*y* HMI/*z* TPOAC/45 H_2_O, where *x* = 0.1 or 0.15; *y* = 0.35 or 0.5, and *z* = 0.03 or 0.04. The reaction mixture was transferred into a 50 ml Teflon-lined stainless steel autoclave, which was rotated at 60 r/min and heated at 150°C for the required time. The conventional MCM-22 and MCM-56 was synthesized according to the reported procedure in the literature [17].

### Material characterizations

XRD patterns were obtained using X-ray diffractometer (SmartLab SE, Rigaku) at 40 kV and 40 mA. N_2_ adsorption-desorption isotherms were measured with Micromeritics ASAP 2460 instrument at −196°C. TEM micrographs were obtained on FEI Tecnai G2 F20 S-TWIN at an acceleration voltage of 200 kV, and SEM observations were performed on Carl Zeiss Sigma with a field-emission gun operated at 5.0 kV. ^27^Al and ^29^Si MAS NMR experiments were performed on Bruker AVANCE III 600 spectrometer at a resonance frequency of 156.4 MHz and 119.2 MHz, respectively. NH_3_-TPD measurements were performed using a BELCAT II (MicrotracBEL) instrument. Chemical compositions were determined with ICP-OES on an Optima 7000DV (PerkinElmer) spectrometer. FTIR spectroscopy with 2,6-di-tert-butyl-pyriding (DTBP) adsorption were obtained on Bruker Vertex 70 instrument. The band at 1615 cm^−1^ corresponding to the protonated DTBP was used to estimate the amount of external Brønsted acid sites.

### Catalytic measurements

The reaction of alkylation of benzene with 1-dodecene was performed in a continuous flow fixed-bed reactor. Typically, the catalyst samples (0.25 g catalyst (40–60 mesh) diluted with quartz sands) were loaded into the reactor, and activated *in situ* at atmospheric pressure in a flow of pure N_2_ (30 cm^3^/min) at 300°C for 4 h. After this, the reactor temperature was decreased to 78°C and the benzene and 1-dodecene (benzene/1-dodecene = 20 : 1) was fed into the reactor under the reaction conditions of atmospheric pressure, 78°C and a space velocity of 2 h^−1^. The conversion was calculated based on the mole of reacted 1-dodecene compared to the mole of 1-dodecene in the feed.

## Supplementary Material

nwaa236_Supplemental_File
