# Supplementary material for: Controlled direct synthesis of single- to multiple-layer MWW zeolite
Source: Natl Sci Rev. 2020 Sep 14;8(7):nwaa236. doi: 10.1093/nsr/nwaa236 (PMC8310756; doi:10.1093/nsr/nwaa236)
Supplement: nwaa236_Supplemental_File [file nwaa236_supplemental_file.docx]

Supplementary data

Controlled Direct Synthesis of Single- to Multiple-layer MWW Zeolite

Jie-Qiong Chen^1^, Yu-Zhao Li^1^, Qing-Qing Hao^1,^*, Huiyong Chen^1^, Zhao-Tie Liu^2^, Chengyi Dai^1^, Jianbo Zhang^1^, Xiaoxun Ma^1^, and Zhong-Wen Liu^2,^*

^1^ School of Chemical Engineering, Northwest University, Chemical Engineering Research Center of the Ministry of Education for Advanced Use Technology of Shanbei Energy, International Science & Technology Cooperation Base of MOST for Clean Utilization of Hydrocarbon Resources, Xi’an 710069, China

^2^ Key Laboratory of Syngas Conversion of Shaanxi Province, School of Chemistry & Chemical Engineering, Shaanxi Normal University, Xi’an 710119, China

***Table of Contents***

**1. Experimental Procedures**

**2. Supporting Scheme and Figures**

**3. Supporting Tables**

1. **Experimental Procedures**

**1.1 Synthesis of MWW zeolites**

**The synthesis of SL-MWW and ML-MWW.** SL- to ML- MWW zeolites were prepared by using dual organic templates, specifically, HMI as the SDA for directing the formation of MWW unit cell and commercially available TPOAC as the assisted SDA to preventing their stacking along *c*-axis. Typically, colloidal silica was added to an aqueous solution of sodium aluminate and NaOH. After stirring the mixture at room temperature for 30 min, the HMI and TPOAC were added to give a gel molar composition of *x* Na_2_O/1 SiO_2_/0.033 Al_2_O_3_/*y* HMI/*z* TPOAC/45 H_2_O, where *x* = 0.1 or 0.15; *y* = 0.35 or 0.5, and *z* = 0.03 or 0.04. The gel was allowed to age under stirring for 2 h at room temperature. Finally, the reaction mixture was transferred into a 50 ml Teflon-lined stainless steel autoclave, which was rotated at 60 r/min and heated at 150 ^o^C for a given period. After quenching the reaction mixture with cold water, the product was filtered, washed with deionized water until pH<9, and dried at 120 ^o^C for 12 h. The as-synthesized material was calcined in air at 550 ^o^C for 6 h.

**The synthesis of MCM-22.** MCM-22 zeolite with SiO_2_/Al_2_O_3_ ratio of 30 was synthesized by hydrothermal crystallization according to the reported procedure. Typically, 0.26 g of sodium aluminate and 0.25 g of NaOH are dissolved in 25.16 g deionized water. Then, 4.67 g of colloidal silica was added to the solution while stirring. The mixture was stirred for 30 mins, and 1.54 g of HMI are added dropwise to the gel. The mixture with a molar Na_2_O/SiO_2_/Al_2_O_3_/HMI/H_2_O composition of 0.15:1.00:0.03:0.5:45.00 was allowed to age under stirring for 30 mins at room temperature. Finally, the reaction mixture was transferred into a 50 ml Teflon-lined stainless steel autoclave, which was tumbled at 60 r/min and heated at 150 ^o^C for 7 days. After quenching the reaction mixture with cold water, the product was filtered, washed with deionized water until pH<9. The MCM-22(P) is dried at 120 ^o^C for 12 h. Direct calcination of the sample in air at 550 ^o^C for 6 h resulted in the products of MCM-22 with the 3D MWW structure.

**The synthesis of MCM-56.** MCM-56 zeolite was synthesized by hydrothermal crystallization according to the reported procedure. Typically, 0.33 g of sodium aluminate and 0.10 g of NaOH are dissolved in 10.95 g deionized water. Then, 4.58 g of colloidal silica was added to the solution while stirring. The mixture was stirred for 30 min, and 1.06 g of HMI are added dropwise to the gel. The mixture with a molar Na_2_O/SiO_2_/Al_2_O_3_/HMI/H_2_O composition of 0.11:1.00:0.04:0.35:19.90 was allowed to age under stirring for 30 min at room temperature. Finally, the reaction mixture was transferred into a 50 ml Teflon-lined stainless steel autoclave, which was tumbled at 60 r/min and heated at 143 ^o^C for 60 h. After quenching the reaction mixture with cold water, the product was filtered, washed with deionized water until pH<9. The as-synthesized material is dried at 120 ^o^C for 12 h. Direct calcination of the sample in air at 550 ^o^C for 6 h resulted in the products of MCM-56.

The H-type zeolites was obtained by treating Na-type zeolites with 1 M NH_4_NO_3_ three times at 80 ^o^C for 2 h followed by calcination at 550 ^o^C for 6 h in air.

**1.2 Materials characterization**

XRD patterns were obtained with a step size of 0.02^o^ and speed of 4^o^/min at 40 kV and 40 mA using X-ray diffractometer (SmartLab SE, Rigaku). N_2_ adsorption-desorption isotherms were measured with Micromeritics ASAP 2460 instrument at -196 ^o^C. The total surface area was estimated by the Brunauer-Emmett-Teller (BET) method. The external surface area was estimated from t-plot using the adsorption isotherm. TEM micrographs were obtained on FEI Tecnai G2 F20 S-TWIN at an acceleration voltage of 200 kV, and SEM observations were performed on Carl Zeiss Sigma with a field-emission gun operated at 5.0 kV. ^27^Al and ^29^Si MAS NMR experiments were performed on Bruker AVANCE III 600 spectrometer at a resonance frequency of 156.4 MHz and 119.2 MHz, respectively. ^27^Al MAS NMR spectra were recorded on a 4 mm probe by small-flip angle technique with a pulse length of 0.5 μs (<π/12) and a 1s recycle delay and a spinning rate of 14 kHz. ^29^Si MAS NMR spectra with high-power proton decoupling were recorded on a 4 mm probe with a spinning rate of 10 kHz, a π/4 pulse length of 2.6 μs, and a recycle delay of 100 s. The chemical shifts of ^27^Al and ^29^Si MAS were referenced to 1 mol/L aqueous Al(NO_3_)_3_ and tetramethylsilane (TMS), respectively. NH_3_-TPD measurements were performed using a BELCAT II (MicrotracBEL) instrument. After the adsorption of NH_3_ on the sample, NH_3_-TPD was performed by raising the temperature to 600 °C at a heating rate of 10 °C/min under a He flow of 30 cm^3^/min. Chemical compositions were determined with inductively coupled plasma optical emission spectroscopy (ICP-OES) on an Optima 7000DV (PerkinElmer) spectrometer. FTIR spectroscopy with 2,6-di-tert-butyl-pyriding (DTBP) adsorption were obtained on Bruker Vertex 70 instrument. Prior to FTIR studies, all studied materials were pressed into the form of self-supporting discs (ca. 10 mg/cm^2^) and pretreated in situ in an IR cell at 400 ^o^C under vacuum conditions for 5 h. After cooling to room temperature, the excess of DTBP was adsorbed for 15 min, and then physisorbed molecules were subsequently removed by evacuation at the same temperature for 1 h. The spectrum of the sample was collected with 2 cm^-1^ resolution. The band at 1615 cm^-1^ corresponding to the protonated DTBP was uesd to estimate the amount of external Brønsted acid sites.

**1.3 Friedel-Crafts alkylation of benzene with 1-Dodecene**

The batch reactions of alkylation of benzene with 1-dodecene were carried out in septum‐sealed, thick walled glass tube with magnetic stirring. Typically, the catalyst powder of 0.25 g was added in the mixture of benzene and 1-dodecene (dried by 5A zeolite) under vigorous stirring, and the alkylation reaction was performed under the conditions of 80 ^o^C, benzene/1-dodecene molar ratio = 8:1, zeolite/1-dodecene weight ratio = 0.3, and reaction time of 3 h.

The stability testing was performed in a continuous flow fixed-bed reactor. Typically, the catalyst samples (0.25 g catalyst (40-60 mesh) diluted with quartz sands) was loaded into the reactor, and activated in situ at atmospheric pressure in a flow of pure N_2_ (30 cm^3^/min) at 300 °C for 4 h. After this, the reactor temperature was decreased to 78 °C and the benzene and 1-dodecene (benzene/1-dodecene = 20:1) was fed into reactor under the reaction conditions of atmospheric pressure, 78 ^o^C and a space velocity of 2 h^-1^. The effluent products were analyzed by a GC with a KB-1 capillary column (30 m × 0.25 mm, 0.5 μm) and a flame ionization detector (FID) (GC-9790 Ⅱ, Fuli Chromatographic Analysis Co., Ltd.). The conversion was calculated based on the mole of 1-dodecene reacted compared to the mole of 1-dodecene in the feed.

## Supporting Scheme and Figures

**Scheme S1.** Illustrative scheme for the formation of MWW nanosheets with tunable thickness

The thicknesses and arrangement of MWW layers can be well regulated by simply changing the synthesis conditions especially the ratio of HMI to TPAOC and the alkalinity. In the case of MCM-22(P), the MWW layers were stacked with highly ordered arrangement along *c*-axis under the inductive effect of HMI (Entry a). For the duel-template system with a higher concentration of HMI and/or alkalinity (Entry b and c), the rate for the formation of the single-unit-cell nanosheet was higher than that for the incorporation of TPOAC, which expels the grafting of organosilane to the crystals of MWW layers at the initial stage. As a result, the ML-MWW nanosheets was formed due to inductive effects of HMI through forming strong hydrogen bonds with surface silanols, the exact number of which is dependent on the synthesis conditions. With an optimal concentration of HMI and alkalinity, the rates between the nucleation/growth of the MWW nanocrystals and the incorporation of the organosilane into the MWW monolayer was matched very well (Entry d), the SL-MWW with ‘house of cards’ was obtained. If the consentration of TPOAC in the synthesis gel was further decreased, the TPOAC molecules grafted on MWW layers were not uniformly distributed due to its low content (Entry e), leading to the disordered structure with a random translation of adjacent layers.

**Figure S1**. XRD patterns for MCM-56 (a), as-synthesized (b) and calcined SL-MWW_0.1/0.35_ (c) with different crystallization time.


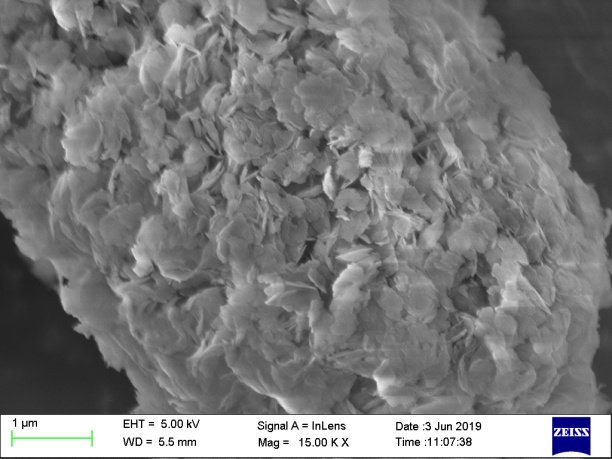




**(c)**

**(b)**

**(a)**







**(d)**







**(f)**

**(e)**

**Figure S2**. SEM images of MCM-22 (a,b), MCM-56 (c,d), and SL-MWW_0.1/0.35_ (e,f)

**Figure S3**. More TEM images of SL-MWW_0.1/0.35_

**Figure S4**. XRD patterns for ML-MWW_0.15/0.5_ with different crystallization time.

**Figure S5**. ^29^Si MAS NMR spectra of as-synthesized samples using a synthesis gel of ML-MWW_0.15/0.5_ with 6 days crystallization (a) and SL-MWW_0.1/0.35_ with 12 days crystallization (b).

**Figure S6**. XRD patterns for ML-MWW_0.1/0.5_ with different crystallization time.

**Figure S7**. XRD patterns for ML-MWW_0.1/0.35/0.03_ (TPOAC/SiO_2_=0.03) with different crystallization time.

**Figure S8**. N_2_ adsorption and desorption isotherms for MCM-22, ML-MWW_0.15/0.5_, ML-MWW_0.1/0.5_, SL-MWW_0.1/0.35_, and ML-MWW_0.1/0.5/0.03_.

**Figure S9.** SEM (top) and TEM (bottom) images of MCM-22 (a, a'), ML-MWW_0.15/0.5_ (b, b'), and ML-MWW_0.1/0.5_ (c, c').

**Figure S10**. ^27^Al MAS NMR spectra of calcined Na-type SL-MWW_0.1/0.35_  before (a) and after (b) heated in 100% steam at 680°C.

**Figure S11**. NH_3_-TPD profiles of MCM-22, MCM-56, and SL-MWW_0.1/0.35_.

**3. Supporting Tables**

**Table S1**. Composition and acidic properties of the MWW zeolites

| Samples | SiO_2_/Al_2_O_3_^[a]^ | Total acidic sites^[b]^  (mmol/g) | External Brønsted sites^[c]^  (mmol/g) |
| --- | --- | --- | --- |
| MCM-22 | 23 | 1.125 | 0.250 |
| MCM-56 | 18 | 1.102 | 0.253 |
| SL-MWW_0.1/0.35_ | 29 | 0.649 | 0.464 |

[a] SiO_2_/Al_2_O_3_ molar ratio measured by ICP-OES

[b] As determined from NH_3_-TPD results

[c] As determined from FTIR spectroscopy with DTBP adsorption

**Table S2**. Catalytic performance of MWW zeolites for the alkylation of benzene with 1-dodecene

| Sample | Conversion of 1-dodecene (%) | TON | Selectivity of LAB  (%) | Product distribution (%) | | | | |
| --- | --- | --- | --- | --- | --- | --- | --- | --- |
|  |  |  |  | 2-P | 3-P | 4-P | 5-P | 6-P |
| MCM-22 | 55.0 | 10.2 | 97.3 | 56.9 | 32.8 | 4.9 | 3.0 | 2.3 |
| MCM-56 | 65.0 | 11.9 | 96.9 | 62.6 | 31.5 | 3.3 | 1.5 | 1.1 |
| SL-MWW_0.1/0.35_ | 63.0 | 21.3 | 97.4 | 59.7 | 28.6 | 5.6 | 3.5 | 2.5 |

Reaction conditions: T=80 ^o^C, t=3 h, catalyst=0.25 g, benzene/1-dodecene molar ratio = 8:1
